# Melt Pool Simulation of Dual Laser Beam-Arc Hybrid Welding of Aluminum Alloy Using Finite Element Method

**DOI:** 10.3390/ma18010135

**Published:** 2024-12-31

**Authors:** Qing-Ye Jin, Jongwook Jung, Jooyong Cheon, Changwook Ji, Wookjin Lee

**Affiliations:** 1 School of Materials Science and Engineering, Pusan National University, Busan 46241, Republic of Korea; sub6953@pusan.ac.kr (Q.-Y.J.); jks3427@pusan.ac.kr (J.J.); 2 Smart Forming Process Group, Korea Institute of Industrial Technology (KITECH), Ulsan 44413, Republic of Korea; cjy0328@kitech.re.kr

**Keywords:** laser-arc hybrid welding, dual laser beam, FEA, thermal analysis simulation, aluminum alloy

## Abstract

In this study, the melt pool formation behavior of high-speed laser-arc hybrid welding of aluminum plates was simulated using finite element analysis (FEA). To evaluate the heat input efficiencies of the laser and arc, standalone laser or arc welding experiments were conducted using the same arc or laser processing parameters as those employed in hybrid welding. These experiments were also simulated using FEA to calibrate the laser and arc heat adsorption parameters. The melt pool shapes were measured from cross-sectional optical microscope (OM) images of the specimens and subsequently used to develop a thermal analysis simulation of the laser and arc welding processes. A simulation model for the laser-arc hybrid welding process was developed by combining the heat input models of the laser and arc welding processes. The FEA model successfully predicted the melt pool shapes observed in the experiments. The accuracy of the developed model was evaluated, yielding average errors in the melt pool sizes of the laser, arc, and hybrid welds of 5.43%, 6.89%, and 4.51%, respectively.

## 1. Introduction

Aluminum alloys are among the most widely used non-ferrous metal alloys, finding applications in various fields, including aerospace, automobiles, and consumer goods. The manufacturing processes for aluminum alloys have undergone significant advancements over time, driven by their distinctive characteristics such as low density, low boiling point, and high chemical reactivity. These characteristics make manufacturing technology critical to achieving high production efficiency and product quality [1,2]. Welding is one of the most important manufacturing techniques for aluminum alloys since most aluminum products require welding [3,4].

The unique characteristics of aluminum alloys, including high thermal conductivity and low boiling point, make their welding process more challenging compared to conventional metal alloys, such as steels [5,6,7]. Research on the welding of aluminum alloys has focused on improving both manufacturing efficiency and product quality. For instance, Sharma et al. [8] investigated the mechanical properties and microstructure of resistance spot welding applied to Al6063 alloy. Their study revealed an increase in the hardness of the nugget center from 56 to 61 HRB, along with changes in microstructure, when the heat input was reduced from 10.6 to 6.6 J. Kim et al. [9] experimentally investigated the laser welding characteristics of Al6061-T6 aluminum alloy sheets, achieving weld strength up to 90% of the base metal strength after post-processing. Vijay et al. [10] studied the mechanical properties of Al2024 and Al6063 alloys joined using gas tungsten arc welding, observing a decline in mechanical properties with an increase in the root gap from 0.5 to 1.5 mm.

Aluminum alloys have relatively low heat absorption from infrared laser beams due to their high reflectivity to infrared light [11]. To overcome this challenge, various laser heat sources for welding have been explored. Morimoto et al. [12] studied the application of a blue diode laser with a wavelength of 450 nm to weld copper, a material with reflectivity properties similar to those of aluminum in the infrared range. They demonstrated increased welding speed and penetration depth by introducing a blue laser for pure copper welding. However, this approach is not directly applicable to aluminum welding, as their heat absorption efficiency from the blue diode laser is less than 10%. Bergmann et al. [13] studied the welding characteristics of aluminum alloys using overlapping laser beams with wavelengths of 808 and 980 nm. This technique successfully mitigated welding defects such as hot cracking by reducing the solidification rate.

Laser-arc hybrid welding, an advanced welding technology, utilizes two different heat sources––laser and arc––simultaneously [14,15,16]. In this study, laser-arc hybrid welding was used to enhance the quality of aluminum welding. A dual laser system, consisting of a Gaussian central laser and a ring-shaped laser, was used. These lasers were aligned with the same central point to control the keyhole [17]. The ring-shaped laser preheated the aluminum alloy surface, enhancing heat absorption from the central laser and reducing the generation of keyhole defects.

FEA is a numerical simulation method used to predict various physical properties, including stress, displacement, and temperature [18,19,20]. For the welding of aluminum alloys, Lu et al. [21] studied the thermal flow simulation in oscillating laser welding. Mohd et al. [22] performed a thermal simulation of gas metal arc welding on an aluminum alloy. Qin et al. [23] simulated the MIG arc welding of an aluminum alloy to galvanized steel plates. In the case of laser-arc hybrid welding, Shi et al. [24] configured simulation of laser-arc hybrid welding of AISI 304 stainless steels, with the arc serving as the leading heat source. The simulation model was a three-dimensional transient model that included dynamic heat transfer and fluid flow of molten material. Du et al. [25] developed a simulation of autogenous double-sided butt welding of chromium-molybdenum alloy steel plates for laser-arc hybrid welding process, with an inclined tungsten inert gas welding torch acting as the leading heat source. The simulation model was developed using SYSWeld software, a commercialized tool specialized for welding simulations. However, there are no reports in the literature on FEA-based predictions of melt pool geometry during hybrid laser-arc welding of aluminum alloys.

In this study, laser-arc hybrid welding was performed on a lap joint of aluminum alloys and FEA was conducted to investigate the melt pool geometry. The welding process used in our previous study [26] is considered. Laser–arc hybrid welding was simulated using FEA. Three types of welding experiments were conducted: laser welding, arc welding, and laser-arc hybrid welding. The melt pool sizes were measured from the cross-sectional OM images of the welding area. Subsequently, simulation models of laser and arc welding were developed from individual laser and arc experiments. Finally, the simulation of laser-arc hybrid welding was completed by merging the previous two models. The moving heat source model of hybrid laser-arc welding was developed using multi-purpose general FEA simulation software of ANSYS (Workbench V2024R1). It focused solely on predictions of melt pool shapes without detailed consideration of complex physical phenomena such as liquid fluid flow and solidification. Therefore, the developed simulation model can more efficiently predict the melt pool shape in comparison to the previous models that focused on the liquid flow and solidification [24,25].

## 2. Welding Process

To investigate the welding characteristics of laser-arc hybrid welding, three sets of lap-joint welding experiments were conducted on aluminum alloys using different welding methods: dual laser, arc, and laser-arc hybrid. For consistency, the parameters of the laser and arc heat sources were identical to those used in the hybrid heat sources for the corresponding welding speeds. The experiments were designed considering the applications in automotive subframe parts welding, where cast and extruded aluminum components are welded by laser-arc hybrid welding with a high welding speed of around 1500–2000 mm/min. Therefore, the welding speeds of 1500 mm/min and 2000 mm/min were selected based on the requirements of the final applications. In total, six experiments were conducted. The processing parameters, adopted from a previous study [26] are listed in Table 1.

**Figure 1:**
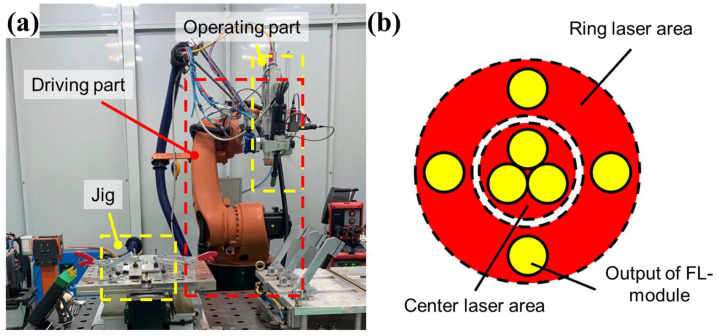
Laser-arc hybrid welding system configuration: (**a**) Jig and welding machine, and (**b**) laser source with multiple fiber laser modules.

The laser-arc hybrid welding machine consisted of a driving part and an operating part, as shown in Figure 1a. The driving module was an industrial robot arm (KUKA AG, Augsbrug, Germany) with six degrees of freedom. The operating part comprised a hybrid welding tip consisting of a laser source and an arc electrode. For the laser source, a Highlight FL-ARM laser (Coherent Inc., Saxonburg, PA, USA) was used. It consisted of seven fiber laser modules (FL-modules) as shown in Figure 1b. The central laser area, comprising three FL-modules, provided a maximum power of 2 kW, whereas the ring-shaped laser area, comprising four FL-modules, provided a maximum power of 4 kW. The arc heat source was a TPS5000 MIG welding machine (Fronius Int’l GmbH, Pettenbach, Austria), capable of delivering a maximum current of 500 A. The constant process parameters used in the welding experiments are listed in Table 2. In laser welding, the focal length of the laser was fixed to 250 mm, for the laser consistently focused on the surface of the samples. In arc welding, the gas flow rate and contact to welding distance (CTWD) were fixed at 15 L/min and 18 mm, respectively, throughout the study. In laser-arc hybrid welding, the laser-arc distance was set to 3 mm, and Argon gas was consistently used as the shielding gas.

**Figure 2:**
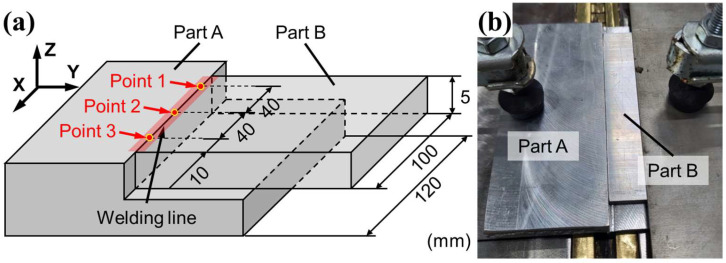
Specimens for laser-arc hybrid welding: (**a**) 3D design and (**b**) assembly fixed by jig.

The specimen used in the experiment was designed to investigate the welding characteristics of hybrid welding under conditions similar to those encountered in the high-speed welding of an automatic component. The sample geometry is shown in Figure 2. Part A of the figure was produced by casting an Al6061 alloy, while Part B was made of extruded Al6063 alloy. Part B (Figure 2a) has a thickness of 5 mm and a length of 100 mm, corresponding to the weld length in the experiment. Part A was designed to be 120 mm in length to fully cover the welding area, marked in red in Figure 2a. The assembly of Parts A and B was fixed using a jig with rubber tips, which applied pressure to the top surface to prevent separation due to thermal distortion during welding, as shown in Figure 2b.

During the welding process, the header of the operating part moved along the welding line in the X-direction. For the laser-arc hybrid welding, the laser beam was focused 3 mm ahead of the arc on the welding line. To investigate changes in the melt pool during welding, three measurement points were set at distances of 40 mm, as shown in Figure 2a. After welding, the specimens were sectioned using wire electrical discharge machining (EDM) to observe the melt pool geometry in the cross sections at the measurement points. To investigate the grain morphology of the sample welded by the laser-arc hybrid welding, the sample welded by the hybrid welding was observed by scanning electron microscope (SEM). The OM and SEM were carried out using Axiolab 5 (ZEISS, Oberkochen, Germany) and JSM7200F (JEOL, Tokyo, Japan), respectively.

**Figure 3:**
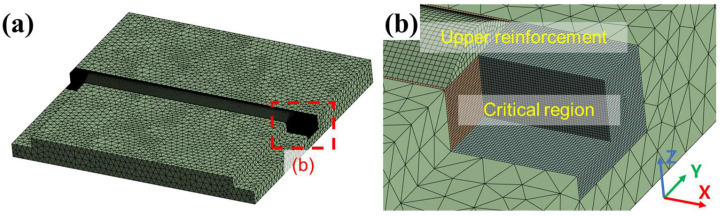
FEA model showing (**a**) overall mesh configuration and (**b**) a detailed view of the highlighted region.

The FEA in this study simulated thermal conduction from multiple heat sources using ANSYS Workbench V2024R1. The computer-aided design (CAD) model for the FEA was designed based on the specimen geometry shown in Figure 3a. For laser welding, the formation of the upper reinforcement was omitted as no filler metal was used. The FEA mesh sizes varied from 0.2 to 2.5 mm to improve calculation efficiency, as shown in Figure 3b. In the critical region, a 10 × 8 mm (width × depth) long bar was located on the welding path. The region was designed to contain all melt pools. The simulation parameters, including those for the boundary conditions and the mesh sizes, are listed in Table 3. The FEA developed in this study focused on thermal conduction analysis and excluded the simulation of material melting and solidification. Material properties, including thermal conductivity, specific heat, and density, were sourced from the ANSYS material library and are listed in Table 4.

**Figure 4:**
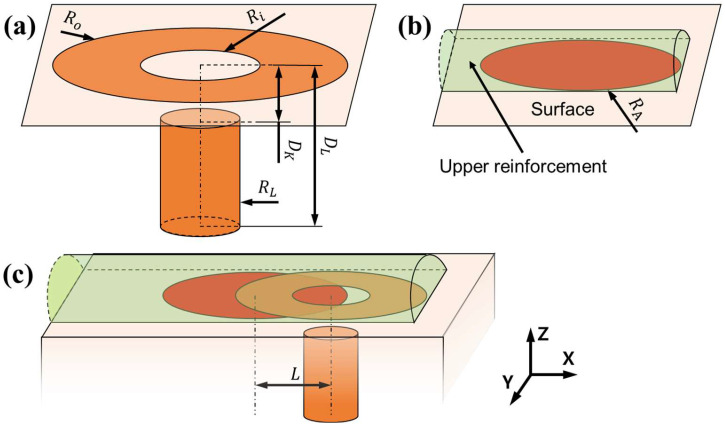
Modeling of heat sources in (**a**) laser, (**b**) arc, and (**c**) laser-arc hybrid welding simulations.

The heat source model for hybrid welding in the FEA simulation was designed by combining the models of the two welding heat sources, laser and arc welding, as shown in Figure 4. For the laser-welding model, the heat source shape was adopted from a previous study [26] and is shown in Figure 4a. It consisted of a central laser area and a ring-shaped laser area. The ring-shaped laser was simulated using node heat input, where nodes on the ring-shaped surface area were selected as target nodes. $R_{o}$ and $R_{i}$ denote the outer and inner radii of the ring, respectively. The central laser area was simulated using element heat input, with the elements inside the cylindrical region selected as the target elements. In the figure, $R_{L}$ denotes the radius of the cylinder. The heat input follows a 2D Gaussian distribution in the x-y plane. Therefore, the heat source of the central laser is represented by a cylinder, as shown in the figure. For the arc welding model shown in Figure 4b, the heat induced by the arc was applied directly to the nodes between the upper reinforcement and welding surface. The nodes inside the arc with a radius of $R_{A}$, were selected as the target nodes, and the heat input was uniformly distributed for each node. In arc welding, the formation of the upper reinforcement was simulated to consider the effect of the filler metal. During FEA, the elements of the upper reinforcement were initially suppressed and progressively unsuppressed as the heat source moved using the element birth and death technique [27].

The nodal heat inputs for the ring-shaped and central laser models were calculated using Equations (1) and (2) as follows:

(1) $$W_{R}^{n}=e_{R}\frac{W_{R}}{{Count}_{R}},$$

(2) $$W_{C}^{e}=e_{C}\frac{W_{C}}{V_{C}}\exp\left(-2\frac{{R_{e}}^{2}}{{R_{L}}^{2}}\right),$$

where $W_{R}^{n}$ is the nodal heat input to the ring-shaped surface, $e_{R}$ is the heat input efficiency of the ring-shaped laser, $W_{R}$ is the total heat input to the ring-shaped laser, and ${Count}_{R}$ is the total number of nodes on the ring-shaped surface. $W_{C}^{e}$ is the heat of the corresponding node of the central laser heat input, $e_{C}$ is the heat input efficiency of the central laser, $W_{C}$ is the total heat input of the central laser, $R_{e}$ is the distance between the corresponding node and the central axis of the cylinder, and $V_{C}$ is the total volume of the cylinder. The volume of the cylinder is calculated using Equation (3), as follows:

(3) $$V_{C}=\pi \left(D_{L}-D_{K}\right){R_{L}}^{2},$$

where $D_{L}$ is the depth of the central laser heat input and $D_{K}$ is the offset between the central laser and the surface, designed considering keyhole generation [28,29].

The nodal heat input for arc welding was calculated using Equation (4):

(4) $$W_{A}^{n}=e_{A}\frac{W_{A}}{{Count}_{A},}$$

where $W_{A}^{n}$ is the nodal heat input in the circular surface, $e_{A}$ is the heat input efficiency of the arc, $W_{A}$ is the total heat input of arc welding, and ${Count}_{A}$ is the total number of nodes within the circular surface.

Laser-arc hybrid welding simulations were performed by combining the laser and arc heat source models, as shown in Figure 4c. In regions where the laser and arc heat sources overlapped, the nodal heat input was calculated as the sum of the individual heat inputs from the two heat source models.

## 3. Experiment Result

**Figure 5:**
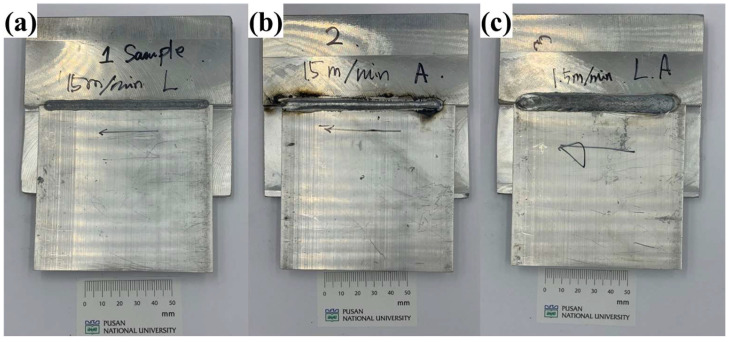
Specimens welded by (**a**) laser welding, (**b**) arc welding, and (**c**) laser-arc hybrid welding.

**Figure 6:**
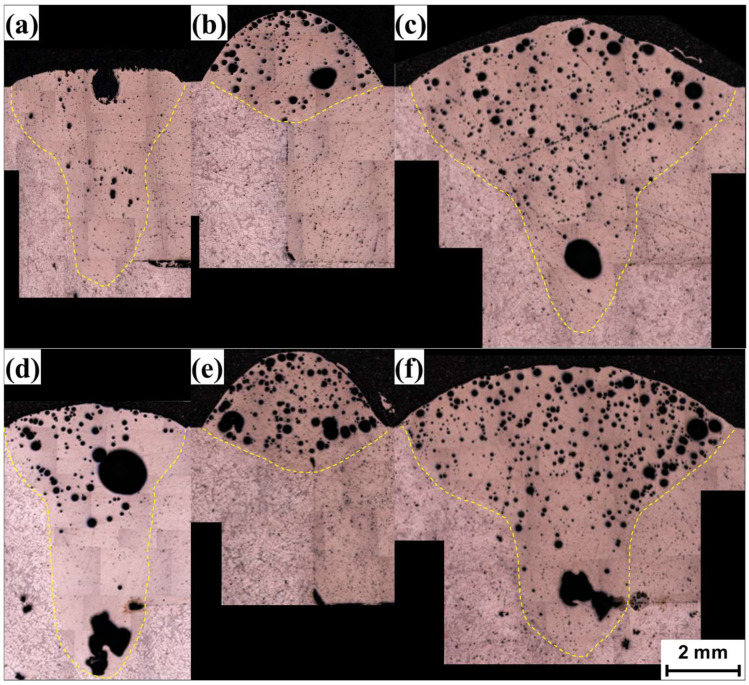
OM images of melt pool at cross-sections corresponding to the measurement points, under welding conditions of (**a**) laser at 1500 mm/min, (**b**) arc at 1500 mm/min, (**c**) hybrid at 1500 mm/min, (**d**) laser at 2000 mm/min, (**e**) arc at 2000 mm/min, and (**f**) hybrid at 2000 mm/min.

The experimental specimens welded using different welding processes are shown in Figure 5. Figure 5a shows the specimen welded exclusively with a dual laser. Figure 5b shows the specimen welded with the arc without a laser, where the upper reinforcement formed by the filler metal is clearly visible. Figure 5c shows the specimen welded using laser-arc hybrid welding, which exhibits a wider upper reinforcement compared to arc welding. To observe the melt pool at the measurement points (Figure 2a), the welded specimens were sectioned using electro discharge machining and then mechanically ground using sandpaper with water. The melt pool images observed through OM of the sectioned specimens are shown in Figure 6.

**Figure 7:**
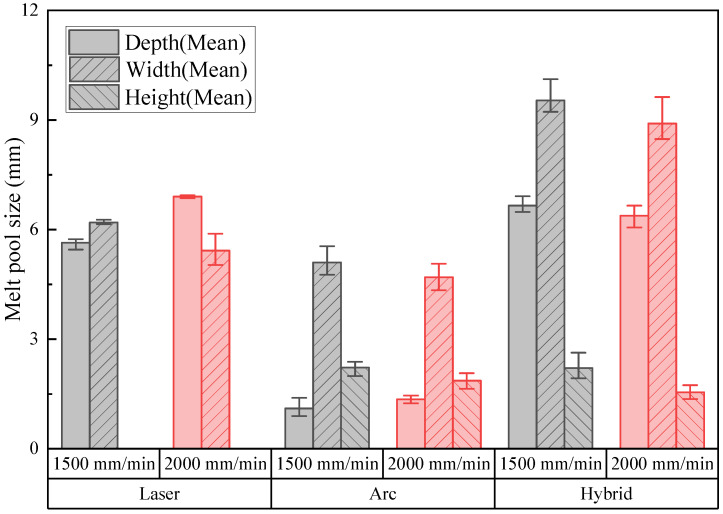
Melt pool sizes measured from the experiment.

The width and depth of the melt pool as well as the height of the filler metal were measured from the OM images and are listed in Figure 7. The melt pool sizes measured at three different points showed no consistent trend with respect to the measurement position. This was probably due to the relatively high welding speed used in this study, which can minimize the melt pool coarsening behavior with welding distance due to heat accumulation. In the figure, the average melt pool sizes for the three measurement points were used as the representative result, and their standard deviations are represented by error bars. A comparison of the melt pools from laser welding (Figure 6a,d) with those from arc welding (Figure 6b,e) shows that the melt pool depths were greater for laser welding, while the widths were similar. Numerous spherical pores were clearly visible in the microscopic images, primarily caused by pitting corrosion of the aluminum alloy during the mechanical grinding process with water. Additionally, irregularly shaped pores, probably resulting from the keyhole effect, were observed. For hybrid welding (Figure 6c,f), the melt pool widths were larger than those observed in laser and arc welding, while the depths were similar to those of laser welding. The filler metals were lower than those observed in arc welding. Comparing the melt pools induced at two different welding speeds of 1500 mm/min (Figure 6a–c) and 2000 mm/min (Figure 6d–f) revealed no significant difference in the melt pool behavior.

**Figure 8:**
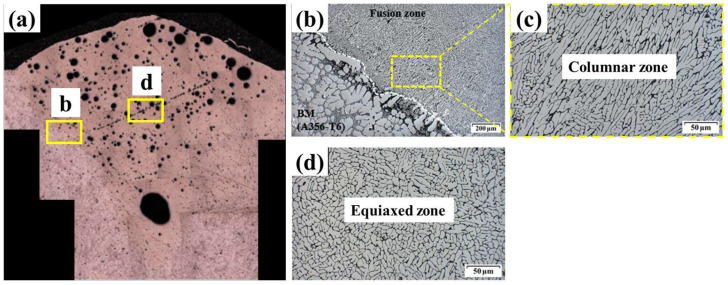
SEM images of (**a**) the sample welded using hybrid welding: (**b**) interface with (**c**) 4× magnification, and (**d**) center of the melt pool.

To investigate the grain morphology of the sample welded by the laser-arc hybrid welding, the interface of the sample welded by the hybrid welding was observed by SEM as shown in Figure 8. The interface observed by SEM was located in the mid-point of the sample which was welded at a welding speed of 1500 mm/min. For the SEM, the sample was polished with ethanol lubricant. In this case, the pores that were previously observed in the OM are no longer observed, indicating that most of the pores observed in the OM image are from the pitting corrosion during the sample preparation. The columnar zone was observed near the melt pool interface as shown in Figure 8a, while the equiaxed zone occupied the remaining melt pool region as shown in Figure 8b. It shows a typical welding microstructure of aluminum, which occurred due to rapid solidification.

## 4. FEA Simulation

**Figure 9:**
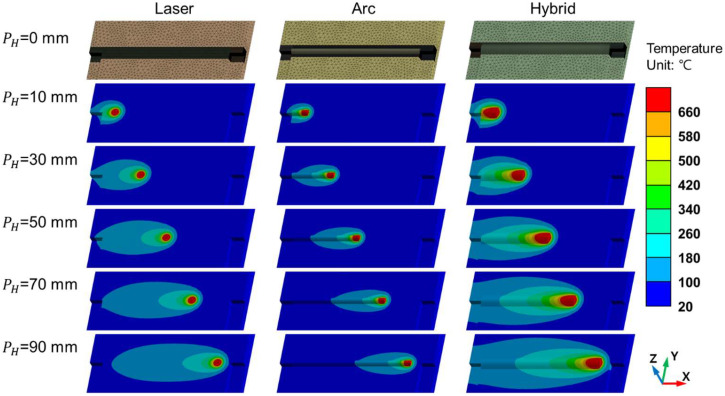
FEA simulations of aluminum alloy welding at different heat source positions ($P_{H}$) for laser, arc, and hybrid welding processes.

The FEA simulations included the entire process of each welding process, including laser, arc, and hybrid welding, as shown in Figure 9. The element used in the model was SOLID278 in ANSYS. The laser welding model included 1,039,670 nodes and 1,226,578 elements. For arc welding, the model at a welding speed of 1500 mm/min contained 1,093,234 nodes and 1,298,031 elements, whereas the model at 2000 mm/min contained 1,092,914 nodes and 1,296,627 elements. For hybrid welding, the model at a welding speed of 1500 mm/min contained 1,073,010 nodes and 1,318,082 elements, and the model at 2000 mm/min contained 1,088,755 nodes and 1,333,431 elements. Each FEA simulation comprised 21 steps, with each step representing a 5 mm displacement of the heat source.

The FEA results were represented by temperature distributions with different colors indicating regions of varying temperatures. The melting temperature of the aluminum alloys was assumed to be 660 °C. In the simulation, areas where the temperature exceeded 660 °C were highlighted in red, representing the molten state. The laser welding and arc welding heat source parameters were optimized to represent the experimentally observed melt pool geometries (Table 5). The input parameters included $e_{C}$, $e_{R}$, $R_{i}$, $R_{o}$, $R_{L}$, $D_{L}$, $D_{K}$, $e_{A}$, and $R_{A}$. The parameters of the heat input model geometry included $R_{i}$, $R_{o}$, $R_{L}$, $D_{L}$, $D_{K}$, and $R_{A}$. These parameters controlled the width-to-depth ratio of the melt pool geometry in the simulations. These were adjusted to reflect the actual geometries of the melt pool shapes observed in the experiments. During the optimization process, the input parameters of $R_{i}$, $R_{o}$, $R_{L}$, $D_{L}$ and $D_{K}$ were fixed parameters. These parameters were adopted from the previous work [26]. The other parameters were optimized to reflect the observed melt pool shapes the best. In the laser welding model, $e_{C}$ and $e_{R}$ were independently adjusted from 100% to 50% in steps of 2.5% to optimize the melt pool depth and width. In the arc welding model, $e_{A}$ was varied from 100% to 50% in steps of 2.5% to optimize the melt pool width. Then, $R_{A}$ was varied from 1 mm to 3 mm in steps of 0.1 mm to optimize the width-to-depth ratio of melt pool geometry. Finally, $e_{A}$ was optimized in the same way to reach the optimized melt pool size. The optimized input parameters of the FEA simulation for both the laser-only and arc-only welding simulations are shown in Table 5.

**Figure 10:**
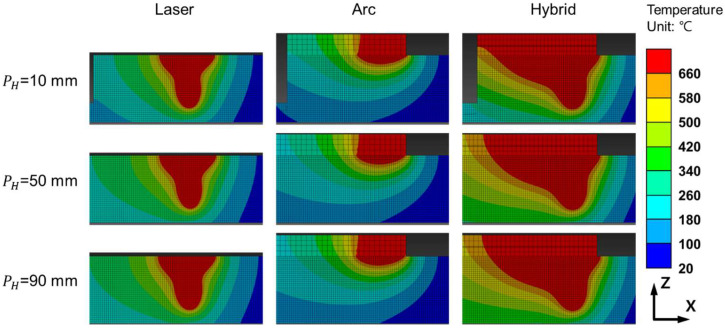
Z-X cross-sectional images of the FEA aluminum alloy welding simulations at different heat source positions ($P_{H}$) for laser, arc, and hybrid welding processes.

**Figure 11:**
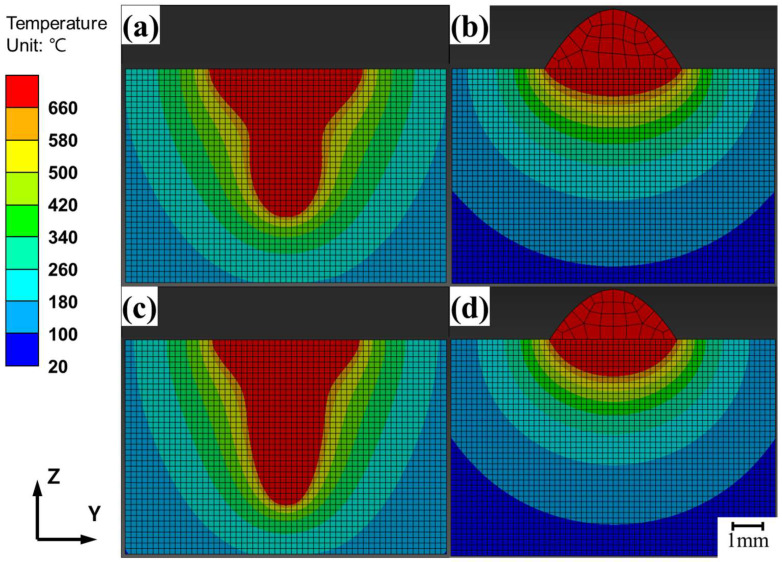
Thermal FEA results of aluminum alloy welding under the following conditions: (**a**) Laser at 1500 mm/min, (**b**) arc at 1500 mm/min, (**c**) laser at 2000 mm/min, and (**d**) arc at 2000 mm/min.

**Figure 12:**
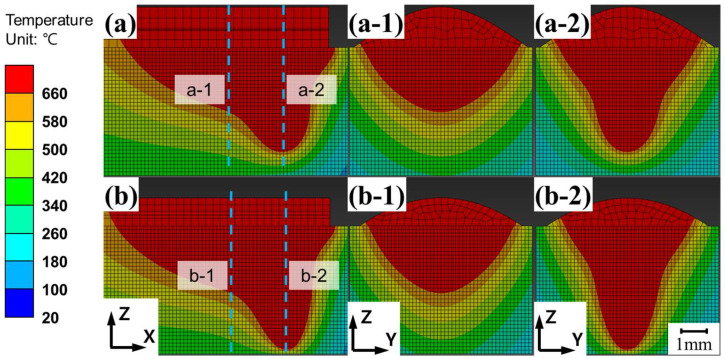
FEA results for laser-arc hybrid welding showing: (**a**) Z-X cross-section at 1500 mm/min; (**a-1**) the Z-Y cross-section at the laser heat input axis; (**a-2**) the Z-Y cross-section at the arc heat input axis; (**b**) the Z-X cross-section at 2000 mm/min; (**b-1**) the Z-Y cross-section at the laser heat input axis; and (**b-2**) the Z-Y cross-section at the arc heat input axis.

Figure 10 shows the z-x cross-section of the FEA results at the measurement points, where the melt pool geometries, indicated in red, vary depending on the heat source. No pronounced differences in the melt pool geometry were observed as the heat source traveled, indicating that the welding speeds used in the experiments were sufficiently high to minimize the heat dissipation effects influencing the melt pool shape during welding for the simulations. Figure 11 and Figure 12 show the Z-Y cross-sections of the FEA results.

The FEA results for laser and arc welding are shown in Figure 11, displaying the Z-Y cross-section at the midpoint ($P_{H}$ = 50 mm) of the heat source path, as shown in Figure 9. The FEA results for laser welding at speeds of 1500 mm/min and 2000 mm/min are shown in Figure 11a,c, respectively, whereas the results for arc welding at the same speeds are shown in Figure 11b.d. The FEA results for hybrid welding are shown in Figure 12. The melt pool geometries differed significantly between laser and arc welding, resulting in a loss of symmetry along the Z-axis in the Z-X cross-section. The Z-X cross-section of the 1500 mm/min hybrid welding FEA result is shown in Figure 12a, the Z-Y cross-section at the laser center axis is shown in Figure 12(a-1), and the cross-section at the arc center axis is shown in Figure 12(a-2). The Z-X cross-section of the 2000 mm/min hybrid welding FEA result is shown in Figure 12b, the Z-Y cross-section at the laser center axis is shown in Figure 12(b-1), and the cross-section at the arc center axis is shown in Figure 12(b-2). The maximum width and depth were located at different Z-Y cross-sections, as shown in the figure.

**Figure 13:**
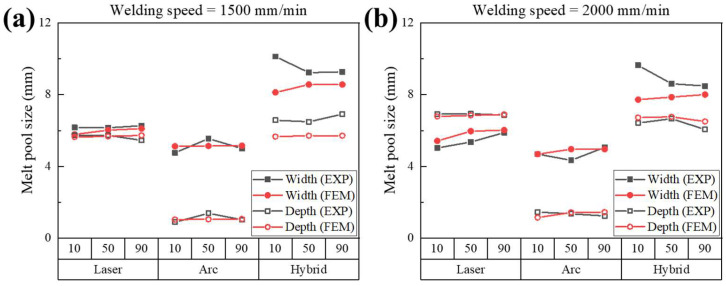
Comparison of experimental and simulated melt pool sizes by welding method and heat source position ($P_{H}$) at welding speeds of (**a**) 1500 mm/min and (**b**) 2000 mm/min.

The melt pool sizes obtained experimentally and those calculated by FEA simulations are presented in Figure 13. The data were categorized by welding speed, welding method, and heat source position ($P_{H}$). For a welding speed of 1500 mm/min (Figure 13a), the average error rates were calculated as 2.21% for laser welding and 2.68% for arc welding. For a welding speed of 2000 mm/min (Figure 13b), the average error rates for the laser and arc welding simulations were 3.91% and 2.01%, respectively.

For hybrid welding simulations, the error rates were slightly higher than those for laser and arc simulations. The average error rates for the hybrid welding simulation were calculated as 7.20% and 5.28% at speeds of 1500 mm/min and 2000 mm/min, respectively. In general, the simulated widths were underestimated compared to the corresponding experimental data at speeds of 1500 and 2000 mm/min. At a speed of 1500 mm/min, the simulation underestimated the depth, while at 2000 mm/min, it slightly overestimated the depth. The largest width discrepancy occurred at $P_{H}=$ 10 mm, and the largest depth discrepancy occurred at $P_{H}=$ 90 mm.

## 5. Discussion

**Figure 14:**
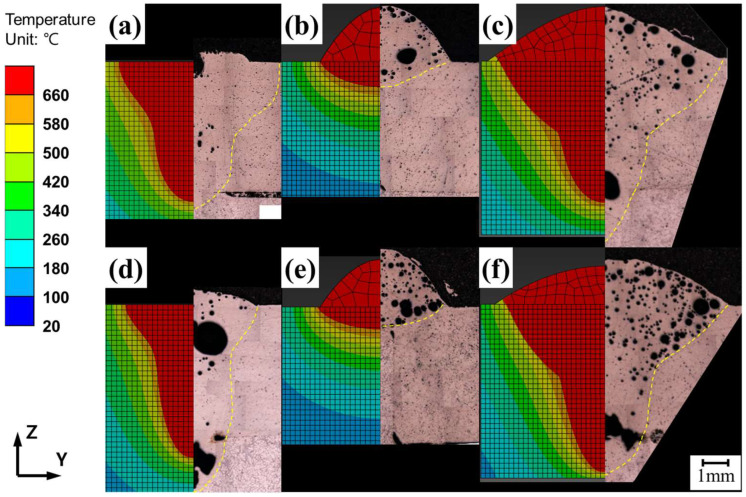
Comparison of experimental and FEA-simulated melt pool images for the conditions: (**a**) laser at 1500 mm/min, (**b**) arc at 1500 mm/min, (**c**) hybrid at 1500 mm/min, (**d**) laser at 2000 mm/min, (**e**) arc at 2000 mm/min, and (**f**) hybrid at 2000 mm/min.

The images of the Z-Y cross-sectional melt pool geometries obtained from the FEA simulations (Figure 11 and Figure 12) were compared with the experimental melt pool images observed via OM (Figure 6), as shown in Figure 14. Figure 14a,b compare the melt pool geometries for laser- and arc-only welding at 1500 mm/min, respectively. Figure 14c shows hybrid welding at 1500 mm/min. Similarly, Figure 14d–f compare the corresponding welding processes––laser, arc, and hybrid––at a welding speed of 2000 mm/min.

The optimization of hybrid welding FEA depends on accurately simulating both laser and arc welding, taking into account variations in welding speed and heat source position. For laser welding, the FEA model was well-optimized, as shown in Figure 14a,d. The effectiveness of the proposed simulation models was evaluated based on the error rates in the weld penetration depth and the width of the melt pool geometry, as well as the width of the lower area and the depth of the upper area of the melt pool, consistent with a previous study [26].

Parameter optimization in the laser welding simulation revealed that the heat input efficiency of the center laser increased with welding speed. This finding reflects the influence of welding speed on laser penetration in the aluminum alloy, which alters the heat source geometry due to keyhole formation. Dynamic changes in the keyhole significantly affect melt pool geometry [30]. Therefore, the heat input efficiency of the central laser had different values in the simulation depending on the welding speed, even though it was fixed in the experiments. The optimized FEA results for laser welding are shown in Figure 14a,d. For arc welding, as illustrated in Figure 14b,e, the FEA results demonstrated a degree of agreement with the experimental results comparable to that observed for laser welding.

The formation of the upper reinforcement during arc welding was simulated using FEA by incorporating the filler metal effect. To simplify the simulation, in this study, the arc heat input was assumed to act on the circular interface between the upper reinforcement and the melt pool, with the $R_{A}$ value representing the upper reinforcement width observed experimentally. Before heat generation, the elements representing the upper reinforcement were formed over the FEA nodes, where arc-induced heat was applied. During heating, the upper reinforcement absorbed a portion of the heat. Therefore, the sizes of the melt pool and the upper reinforcement are closely interrelated due to the heat dissipation in two different directions to the reinforcement and melt pool, as observed in the FEA simulations.

The melt pool geometries for hybrid welding differed significantly from those of the other welding processes in the FEA simulations, as illustrated in Figure 14. The Z-Y cross-sections of the melt pool in the hybrid welding simulation (shown in Figure 14c,f) were generated by overlapping the Z-Y cross-sections of the melt pool geometry along the axis of the laser heat input and the arc heat input (Figure 12). The images used for overlapping were selected from the sections where the maximum weld penetration depth and melt pool width were observed. The average error rate for the hybrid welding simulation was 6.2%, which is comparable to the previous simulation works by Shi et al. [24] and Du et al. [25], where average error rates of 4.7% and 8.1%, respectively, were obtained. This suggests that the developed simulation model is able to predict similarly accurate results in the prediction of melt pool shape in comparison to the previous models that considered complex liquid flow, even though the current model did not consider directly the liquid flow behavior.

The hybrid welding simulation was configured by combining the heat input parameters optimized from individual laser and arc welding experiments. Although the parameter sets for the laser-arc hybrid welding experiments were not individually optimized, simulations reproduced the melt pool shapes of the experiments reasonably well. This implies that the laser and arc heat inputs in hybrid welding are comparable to those in laser- or arc-only welding. A study by Liu et al. [31] suggested that the laser-induced plasma in the keyhole enhances the electrical conduction in the electric arc, providing additional charge carriers during the interaction between the laser and arc. This complex phenomenon has not been considered in the current FEA model. If additional heat absorption occurs during hybrid welding, the experimentally observed melt pool sizes are expected to exceed those predicted by simulations. According to the results shown in Figure 14c,f, additional heat absorption was observed at a welding speed of 1500 mm/min, leading to slight discrepancies between the experimental and simulated melt pool shapes. However, this effect was less pronounced at a welding speed of 2000 mm/min. The discrepancy between the experiments and the simulations decreased from 7.2% to 5.3% as the welding speed increased from 1500 mm/min to 2000 mm/min. This indicates a reduction in additional heat absorption with the increase in welding speed. This was probably due to the fact that the additional heat absorption decreases as the laser-arc interaction time shortens with higher welding speed.

## 6. Conclusions

In this study, an FEA model for laser-arc hybrid welding was developed by combining individually optimized FEA models for laser and arc welding. Lap-joint welding experiments were conducted for FEA model parameter optimization using three welding processes: laser, arc, and laser-arc hybrid. The findings from FEA simulations and experimental data are summarized as follows:

1. The FEA model for laser welding was optimized to achieve an average error rate of 3.06%, whereas the FEA model for arc welding achieved an average error rate of 2.36%.
2. The average error rates for the FEA model of hybrid welding, which combines the laser and arc welding models, were 7.20% and 5.28% for welding speeds of 1500 mm/min and 2000 mm/min, respectively.
3. In the FEA results for laser-arc hybrid welding, the maximum width and depth of the melt pool geometry did not occur in the same Z-Y cross-section. They were located in sections corresponding to the center axes of the arc and laser heat sources. The final melt pool shapes predicted by intersecting the melt pools caused by the laser and the arc in the simulations correspond very well to the experimentally observed shapes.
4. Additional heat absorption due to the laser-arc interaction was observed when the welding speed was 1500 mm/min, as confirmed by comparing the FEA and experimental results. The additional heat absorption was thought to be from the laser-arc interaction. This effect was decreased at a welding speed of 2000 mm/min, probably due to the fact that the additional heat absorption decreases as the laser-arc interaction time shortens with higher welding speed.

The FEA simulation developed in this study shows a large potential for predicting the melt pool shape and overall temperature distribution in the applications of laser-arc hybrid high-speed aluminum alloy welding. In future research, the impact of the laser-arc distance on melt pool formation will be investigated. The additional heat absorption caused by the interaction between the laser and arc is closely related to the overlapping region of the heat sources. However, this overlapping region has not been explicitly modeled in the FEA simulation. The laser-arc distance is considered a critical parameter to determine the overlapping region and, consequently, the additional heat absorption due to the laser-arc interaction. This will be studied in the near future to understand the physical phenomena occurring in the laser-arc hybrid welding process.

## Figures and Tables

**Table 1:** Parameters for laser-arc hybrid welding of aluminum alloy.

Welding Speed (mm/min)	Welding Method	Laser Parameter		Arc Parameter	
		Center (kW)	Ring (kW)	Current (A)	Voltage (V)
1500	Laser	2.0	3.0	-	-
	Arc	-	-	150	21.2
	Hybrid	2.0	3.0	150	21.2
2000	Laser	2.0	4.0	-	-
	Arc	-	-	170	21.7
	Hybrid	2.0	4.0	170	21.7

**Table 2:** Contant process parameters of welding experiments.

Laser		Arc		Hybrid	
Focal Length (mm)	Defocus Distance (mm)	Gas Flow Rate (L/min)	CTWD (mm)	Laser-Arc Distance (mm)	Shielding Gas
250	0	15	18	3	Ar

**Table 3:** Parameters for the configuration of laser-arc hybrid welding simulation.

The Film Coefficient (W/mm^2^ °C)		Ambient Temperature (°C)		Mesh Size (mm)		
Bottom	Surface	Bottom	Surface	Critical Region	Upper Reinforcement	Default
$1.2\times 10^{-3}$	$5.0\times 10^{-6}$	90	22	0.2	0.4	2.5

**Table 4:** Material properties for laser-arc hybrid welding of aluminum alloy.

Temperature (°C)	−100	0	100	200
Thermal conductivity (W/m °C)	114	144	165	175
Density (g/cm^3^)	2.77	2.77	2.77	2.77
Specific heat (J/kg °C)	875	875	875	875

**Table 5:** Optimized parameters for FEA simulations of laser and arc welding.

Welding Speed (mm/min)	Laser Parameter							Arc Parameter	
	Optimized		Fixed					Optimized	
	$e_{C}$ (%)	$e_{R}$ (%)	$R_{i}$ (mm)	$R_{o}$ (mm)	$R_{L}$ (mm)	$D_{L}$ (mm)	$D_{K}$ (mm)	$e_{A}$ (%)	$R_{A}$ (mm)
1500	80.0	63.3	0.5	2.5	0.4	5.2	3	62.9	2.5
2000	95.0	60.0	0.5	2.5	0.4	6.5	2.2	55.6	1.9

## Data Availability

The datasets presented in this article are not readily available because the data are part of an ongoing study. The raw data supporting the conclusions of this article will be made available conditionally by the authors on request.
